# Defective I1 dynein in *Chlamydomonas* axonemes is epistatic to the RII-binding domain function of radial spoke protein 3 (RSP3) in the regulation of ciliary motility.

**DOI:** 10.17912/micropub.biology.001890

**Published:** 2025-12-19

**Authors:** Martin J Sebastian, Ashley Solmonson, Anne R Gaillard

**Affiliations:** 1 Department of Biological Sciences, Sam Houston State University, Huntsville, Texas, United States

## Abstract

In *Chlamydomonas* , the central pair (CP) and radial spoke (RS) complexes in the axoneme are key regulators of ciliary motility. Radial spoke protein 3 (RSP3) is an A-kinase anchoring protein (AKAP), and mutation of the RII-binding domain ( *388* ) results in specific ciliary motility defects. When combined with *ida1* , a mutant defective in the 1α-dynein heavy chain required for assembly of the I1 dynein complex, the phenotype of the resulting *388; ida1* double mutant is *ida1* -like, not *388* -like; thus, *ida1* is epistatic to *388* . These results support I1 dynein being downstream of the RSP3 RII-binding domain function in a signaling pathway that regulates *Chlamydomonas* ciliary motility.

**Figure 1. Phenotypic analysis of 388 , ida1 , and 388 ; ida1 mutants compared to wild-type:**
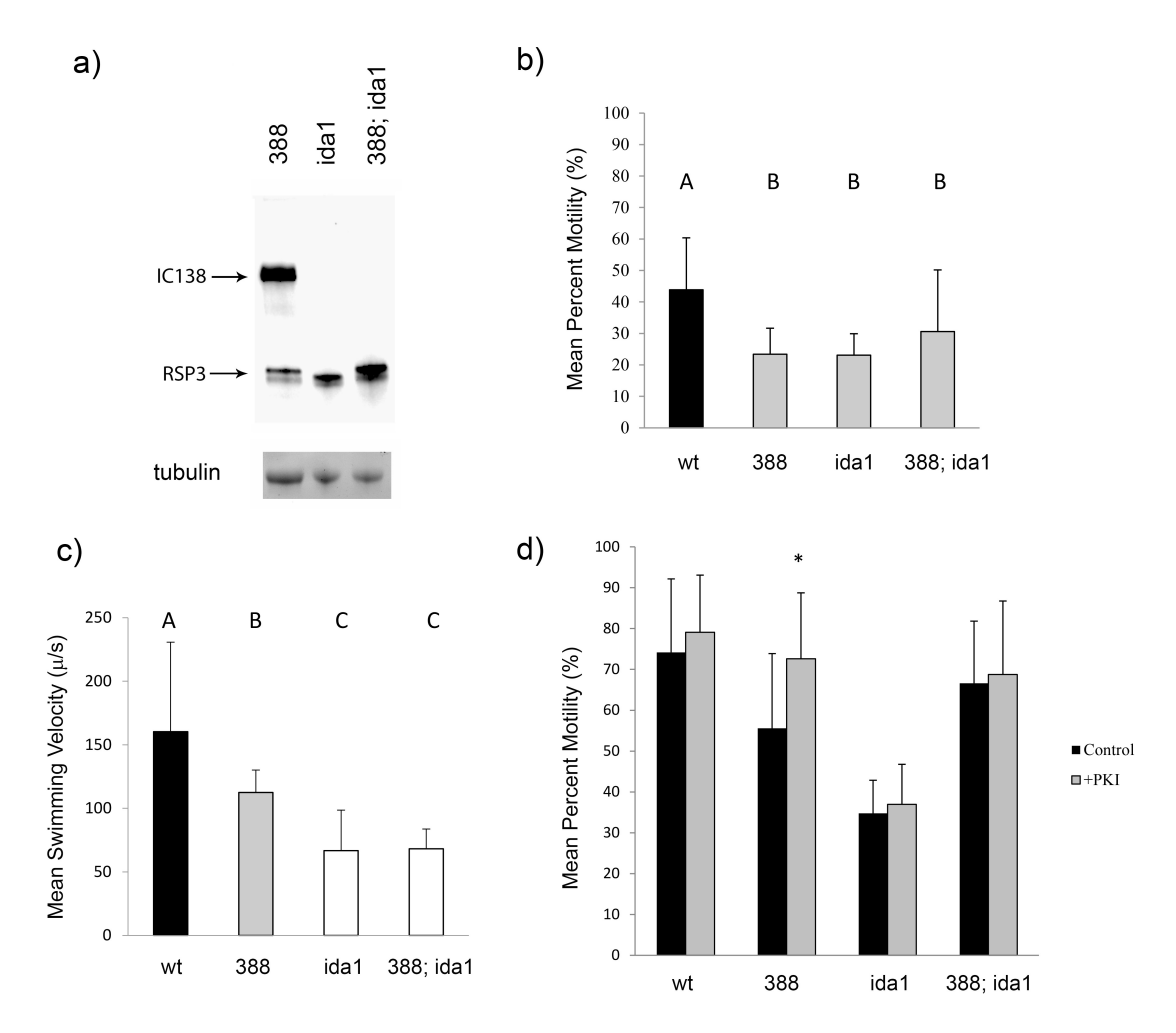
a) Verification of the *388; ida1* double mutant strain. Axonemal protein (40 μg) of, *388* , *ida1* , and *388; ida1* strains was separated by SDS-PAGE using a 7.5% polyacrylamide gel. *Above* ) Western blot with co-incubation of α-RSP3 and α-IC138 (a subunit of I1 dynein) antibodies. The *388; ida1* double mutant shows both a slower-migrating version of RSP3 (as in the *388* parent strain) and a lack of IC138 (as in the *ida1* parent strain), thus verifying the double mutant. *Below* ) SDS-PAGE gel section stained with Coomassie blue demonstrating the presence of relatively equal amounts of tubulin, an axonemal protein, in each sample. b) Live Cell Percent Motility. The percentage of motile cells was determined for live populations of wild-type (wt) (N=3), *388* (N=6), *ida1* (N=4), and *388; ida1* (N=6) cells. Any type of cell movement (twitching, spinning, or swimming) was scored as motile. Wild-type cells were significantly more motile than *388* cells (p = 0.0006), *ida1* cells (p = 0.0005), and *388; ida1* cells (p = 0.0415), while all three mutant cell types displayed similar motility when compared to each other (p > 0.05). Tukey post-hoc analysis revealed two significantly different groups overall (A and B). N indicates the number of biological replicates performed for each cell type; each biological replicate consisted of five technical replicates. c) Live Cell Swimming Velocity. Forward progressing swimming velocity was determined for wild-type (wt), *388,* *ida1* , and *388; ida1* cells (N=200 for each cell type). Wild-type cells swam at a significantly higher velocity compared to any of the other strains (p < 0.0001). *388* cells swam at a significantly higher velocity than *ida1* or *388; ida1* cells (p < 0.0001). However, *ida1* cells and *388; ida1* cells swam at similar velocities (p = 0.6866). Tukey post-hoc analysis revealed three significantly different groups (A, B, and C). N indicates the number of biological replicates; each biological replicate consisted of an average of ten technical replicates. d) Reactivation of Motility in Cell Models +/-PKI. Wild-type (wt), *388* , *ida1* , and *388; ida1* cells were demembranated in 0.05% NP-40 to generate cell models with inactivated motility. Motility was reactivated upon the addition of ATP, either in the presence or absence of PKI, a specific inhibitor of PKA. Pair-wise comparisons were performed between cell models with or without PKI, and *388* cells were the only cell type to exhibit statistically greater motility upon the addition of PKI (p = 0.0024; asterisk). All other cell types showed no significant increase in motility in the presence of PKI (p > 0.20). Wild-type, N=8; *388* , *ida1* , and *388; ida1* , N=6. N indicates the number of biological replicates; each biological replicate consisted of five technical replicates.

## Description

In *Chlamydomonas* , the radial spoke (RS) and central pair (CP) complexes regulate the coordinated bending patterns of cilia by controlling dynein-driven microtubule sliding of the doublet microtubules in the axoneme (Smith and Yang, 2004). How these regulatory complexes coordinate to control dynein activity and ciliary motility is still not well understood, but it is clear the regulatory system is multifaceted and complex. Cryo-electron microscopy and tomography studies have revealed key structural components of RS complexes, emphasizing the importance of mechanochemical transducers in the axoneme (Barber et al., 2011; Pigino et al., 2011; Oda et al., 2014; Grossman-Haham et al., 2021; Gui et al., 2021; Zhao et al., 2025). In particular, the axoneme is known to contain many signaling proteins important for regulation of motility, including kinases and phosphatases (Porter and Sale, 2000). One type of signaling protein identified both within the RS and CP of *Chlamydomonas* is an A-Kinase Anchoring Protein (AKAP); AKAPs RSP3 and AKAP240 have been identified in the RS and CP, respectively, and several other AKAPs have been identified in cilia (Gaillard et al., 2001; Kultgen et al., 2002; Carr and Newell, 2007; Bachmann et al., 2016; Rao et al., 2024). AKAPs were originally characterized as a family of proteins that bind to and anchor the regulatory subunits of cAMP-dependent protein kinase (PKA) (Wong and Scott, 2004; Welch et al., 2010). However, the definition of AKAP has since broadened to include the anchoring of a diversity of proteins that contain so called dimerization and docking (D/D) domains (Newell et al., 2008; Sivadas et al., 2012). In addition to AKAPs, homologs of AKAP-interacting proteins have been identified in *Chlamydomonas* axonemes (Yukitake et al., 2002; Dymek and Smith, 2007; Rao et al., 2016).

In *Chlamydomonas* , experimental evidence points to PKA activity being part of a signaling pathway that includes the CP and RS, as well as I1 inner arm dynein (Smith and Sale, 1992; Howard et al., 1994; Habermacher and Sale, 1997; King and Dutcher, 1997; Smith, 2002). Specific to the role of AKAPs in this pathway, a mutant of RSP3 ( *pf14::RSP3 VL→AA _169-170_* ) ( *388* ), in which the amphipathic helix/RII-binding domain has been disrupted, displays abnormal ciliary motility that can be rescued by the addition of PKI, a specific inhibitor of PKA, suggesting that PKA is overactive or mis-regulated when PKA is not properly anchored by RSP3 (Gaillard et al., 2006). This experimental evidence, in combination with other studies suggesting I1 dynein as a substrate of PKA activity (King and Dutcher, 1997), led us to propose a model in which I1 dynein is downstream of a signaling pathway that includes the anchoring and regulation of PKA by RSP3. To test this idea, we combined *388* with a mutation in I1 dynein ( *ida1)* to create *a 388* ; *ida1* double mutant and determined the resulting phenotype.

To validate the *388; ida1* double mutant, a Western blot was performed using axonemal protein extracts and both α-RSP3 and α-IC138 (a subunit of I1 dynein) antibodies (Figure 1a). RPS3 from *388* migrates slightly slower on an SDS-PAGE gel compared with wild-type RSP3 (Gaillard et al., 2006), providing a marker for the *388* protein. Phenotypic analysis was then conducted. First, the percentage of motile cells in populations of each cell type was determined, and average percent motilities of the populations were compared among the different cell types (Figure 1b). As expected, populations of wild-type cells (group A) were significantly more motile than populations of *388* , *ida1* , or *388; ida1* cells (group B; p=0.0006, p=0.0005, p=0.0415, respectively); additionally, the motilities for populations of the three mutant cell types were not significantly different from each other (p > 0.05). While the percentages of motility for populations of both wild-type and *388* cells reported in this study are noticeably lower than in a previously published study (Gaillard et al., 2006), it is important to note that in *both* studies, *388* cells are only about half as motile as wild-type cells. We suggest that the *relative* difference in percent motility between populations of wild-type and *388* cells is of importance here—not the absolute values.

Swimming velocities of cells were also determined. Cells swimming with forward progression (as opposed to spinning or turning cells) from each population were selected, swimming velocity was measured using software analysis, and average swimming velocities for each cell type were compared (Figure 1c). Wild-type cells exhibited a significantly higher average swimming velocity compared to any of the other cell types (group A; p<0.0001), while *388* cells swam with intermediate average velocity (group B), which was significantly faster than either *ida1* or *388; ida1* cells (group C; p<0.0001). While *ida1* and *388; ida1* cells had significantly slower average swimming velocities compared to wild-type or *388* cells, importantly, the average swimming velocities of *388; ida1* and *ida1* cells were insignificantly different from one another (p=0.6866).

To determine whether PKI can rescue the decreased percentage of cell motility observed in populations of *388; ida1* cells, as it has previously been shown to do in populations of *388* cells (Gaillard et al., 2006), cell models for all cell types were generated, and motility was reactivated upon the addition of ATP in either the presence or absence of PKI, a specific peptide inhibitor of cAMP-dependent protein kinase (PKA). For each cell type, average percent motility of the population treated with PKI was compared to the control population for that cell type in which no PKI was added (pair-wise comparison) (Figure 1d). As expected, the addition of PKI significantly increased the average percent motility for populations of *388* cells (p=0.0024) but had no effect on wild-type cells (p=0.2979). In addition, PKI had no significant effect on the motility of *ida1* populations (p=0.3340), and most importantly, PKI was not able to significantly improve motility of *388* ; *ida1* cells (p=0.4593). This observation is consistent with our hypothesis that the target of mis-regulated PKA activity in *388* cells is I1 dynein. Of note, inter-strain comparisons of motility for cell models are not reliable, since in our experience, reactivation of motility for cell models is affected by numerous variables, including age of ATP stock and time of day (circadian rhythm). Care was taken to perform +/- PKI experiments at the same time for each strain.

The results of the above-described experiments are consistent with our model that I1 dynein is downstream of a signaling pathway that includes the anchoring and regulation of PKA by RSP3. However, alternative explanations for our results are also supported. For example, it is possible that a defect in the RII-binding domain of RSP3 results in a deficiency of RS assembly, thus resulting in decreased motility that is characteristic of many *Chlamydomonas* strains deficient in components of the RS. Accordingly, Sivadas et al. (2012) provided evidence that the RII-binding domain of RSP3 interacts with radial spoke proteins 7 (RSP7) and 11 (RSP11) in the RS, suggesting that RSP3 may not anchor PKA in the axoneme at all, but instead may anchor other RS proteins as part of a structural scaffold necessary for RS assembly. However, the aforementioned studies of *388* showed no deficiencies of RSP7 or RSP11 in the RS, and RS appeared to be assembled at wild-type levels. Thus, while it is very unlikely that a mutation in the RII-binding domain of RSP3 leads to a major defect in RS assembly, it is possible that a protein-protein interaction critical for normal motility is lacking in the RS of *388* cells, resulting in a deficiency of signal transmission from the CP through the RS and to I1 dynein. Consistent with this, when RSP11 is defective ( *pf25* ), motility defects occur that closely resemble those of *388* cells (Yang and Yang, 2006).

If we consider that RSP3 may not actually anchor or regulate PKA *directly* in the *Chlamydomonas* axoneme, we must then account for the observation that PKI specifically rescues inhibited motility of *388* cells. One possibility is that a mutation in the RII-binding domain of RSP3 causes a deficiency of protein-protein interaction in the RS, which disrupts signal transduction from the CP through the RS to I1 dynein, leading to overactive PKA elsewhere in the signaling pathway. Overactive PKA may then affect downstream signaling components that in turn inhibit I1 dynein activity. Indeed, evidence shows that a variety of disruptions in the CP-RS-I1 dynein signaling pathway result in overactive PKA. For example, PKI also rescues deficient motility of *Chlamydomonas* mutants defective in the CP ( *pf18* ), or the nearby radial spoke heads of the RS ( *pf17* ) (Habermacher and Sale, 1997), even though normal RSP3 is present in these mutants.

## Methods

***Chlamydomonas Strains and Growth Conditions***

*Chlamydomonas reinhardtii* strains wild type (cc-125) and *ida1* (cc-2664; lacks I1 dynein) were obtained from the *Chlamydomonas* Resource Center (University of Minnesota, St Paul, MN). *388* cells ( *pf14::RSP3 VL→AA _169-170_* ) were previously created as described in Gaillard et al., 2006. Cells were maintained on solid Tris-acetate-phosphate (TAP) medium. Cells were grown in either liquid TAP medium or liquid modified medium I, with aeration and a 14-hr/10-hr light/dark cycle. All media components were obtained from Sigma-Aldrich, with the exception of Bacto™ Agar which was obtained from BD Diagnostics.

***Creation of the ida1; 388 Double Mutant Strain***

*ida1* (mating-type plus) and *388* (mating-type minus) cells were mated in accordance with previously published methods (Gaillard et al., 2006). Progeny cells were screened for the presence of both the *ida1* and *388* mutations by isolating axonemes and performing Western blotting.

***Isolation of Axonemes and Western Blot Analysis***

Cells were grown in liquid TAP medium, and axonemes were isolated as described previously (Habermacher and Sale, 1997) with the exception that the protease inhibitors PMSF and aprotinin were replaced by SIGMAFAST™ Protease Inhibitor Cocktail Tablets (S8830), used at 1 tablet per 100 ml of solution. Western blot analysis was performed by SDS-PAGE separation of axonemal protein using a 7.5% polyacrylamide gel, followed by transfer to a nitrocellulose membrane. The membrane was blocked in 5% nonfat milk in TBS (20 mM Tris, 500 mM NaCl, pH 7.4) for at least 1 h. The membrane was then co-incubated with anti-RSP3 serum at 1:10,000 and anti-IC138 (a component of I1 dynein) serum at 1:20,000 overnight at 4°C. After washing in TBS 3 times, 5 min each, the membrane was incubated in goat anti-rabbit secondary antibodies (1:20,000) (Bio-Rad) for 1 hr at room temperature. The membrane was then washed as previously described and developed using enhanced chemiluminescence (SuperSignal West, Thermo Fisher Scientific).

***Reactivation of Cell Motility***

Cells were grown to an approximate density of 5 x 10 ^6^ cells/ml in liquid modified medium I. Reactivation of cell motility was performed according to previously published methods (Gaillard et al., 2006). All chemicals were obtained from Sigma-Aldrich except for NP-40, which was obtained from Calbiochem.

***Analysis of Cell Motility***

Live or reactivated cells at a density of approximately 5 x 10 ^6^ cells/ml were assessed for motility as described previously (Gaillard et al., 2006). The total number of cells counted for each cell type varied depending on actual cell density but was approximately 1500-3000. For swimming velocity measurements, cells swimming with forward progression were selected from the recordings for measurement of forward progression swimming velocity. Using Metamorph® software (Molecular Devices), swimming velocity was determined by measuring the distance traveled by the cell over time (10 s). Swimming velocities of 100 independent cells were measured for each cell type.

***Statistical Analysis***

SAS statistical software (version 9.4, SAS Institute) was used to perform one-way ANOVA and Cochran non-parametric t-tests. For percent motility, arcsine transformation of the data was performed prior to statistical analysis. Tukey and REGWQ post-hoc analyses were performed to identify significantly different groups.

## Reagents

**Table:** 

Strain	Genotype	Available From
cc-2664	*Chlamydomonas reinhardtii* ; *ida1*	*Chlamydomonas* Resource Center, University of Minnesota
*388*	* Chlamydomonas reinhardtii; (pf14::RSP3 VL→AA _169-170_ ) *	
*388* ; *ida1*	* Chlamydomonas reinhardtii; (pf14::RSP3 VL→AA _169-170_ ) ida1 *	
